# A systematic review and meta analysis on digital mental health interventions in inpatient settings

**DOI:** 10.1038/s41746-024-01252-z

**Published:** 2024-09-17

**Authors:** Alexander Diel, Isabel Carolin Schröter, Anna-Lena Frewer, Christoph Jansen, Anita Robitzsch, Gertraud Gradl-Dietsch, Martin Teufel, Alexander Bäuerle

**Affiliations:** 1 Clinic for Psychosomatic Medicine and Psychotherapy, LVR-University Hospital Essen, University of Duisburg-Essen, Essen, Germany; 2 Center for Translational Neuro- and Behavioral Sciences, University of Duisburg-Essen, Essen, Germany; 3 Department of Child and Adolescent Psychiatry, Psychosomatics and Psychotherapy, LVR-University Hospital Essen, University of Duisburg-Essen, Essen, Germany

**Keywords:** Randomized controlled trials, Psychiatric disorders

## Abstract

E-mental health (EMH) interventions gain increasing importance in the treatment of mental health disorders. Their outpatient efficacy is well-established. However, research on EMH in inpatient settings remains sparse and lacks a meta-analytic synthesis. This paper presents a meta-analysis on the efficacy of EMH in inpatient settings. Searching multiple databases (PubMed, ScienceGov, PsycInfo, CENTRAL, references), 26 randomized controlled trial (RCT) EMH inpatient studies (*n* = 6112) with low or medium assessed risk of bias were included. A small significant total effect of EMH treatment was found (*g* = 0.3). The effect was significant both for blended interventions (*g* = 0.42) and post-treatment EMH-based aftercare (*g* = 0.29). EMH treatment yielded significant effects across different patient groups and types of therapy, and the effects remained stable post-treatment. The results show the efficacy of EMH treatment in inpatient settings. The meta-analysis is limited by the small number of included studies.

## Introduction

Mental health disorders represent a prevalent set of clinical conditions associated with substantial personal and economic burdens. However, despite their prevalence and impact, there exists a conspicuous deficit in the provision of effective treatment^1–4^. Across Europe, estimates suggest that 15–40% of the population experiences some form of mental disorder, yet fewer than one-third of these cases receive treatment that meets the established standards of adequacy^5–9^.

One reason for the lack of adequate treatment of mental disorders are structural supply issues, for example caused by a shortage of mental healthcare providers in more rural areas^10^. Furthermore, negative attitudes towards mental health treatments hinder seeking help especially in mild to moderate cases^11^. Finally, prompt access to mental health treatment is paramount for its efficacy, yet mental health facilities and specialists often impose prolonged waiting periods spanning several months^12^. These extended waiting intervals amplify the economic strain of mental disorders^13^, exacerbate clinical manifestations^14,15^, diminish treatment adherence, and elevate dropout rates^16,17^. In summary, providing adequate mental health treatment is complicated by a variety of structural issues leading to several other problems like economic and patients’ personal costs.

E-mental health (EMH) interventions aim to provide adequate treatment of mental health disorders through technological means and channels, such as app- or web-based systems, text messages, videos, or digital monitoring. Here, the term EMH is used to describe any digitally delivered interventions with the goal of improving mental health outcomes. Due to the easy accessibility of EMH products, such interventions have many advantages: They can 1) fill structural supply gaps for rural areas, 2) bridge long waiting times for in-person mental health treatment, and 3) provide additional anonymity for those concerned about stigmatization^18,19^. Thus, EMH tools have the potential to be a viable method to overcome the various issues hindering adequate mental health treatment.

In outpatient settings, EMH interventions are effective tools to treat mental disorders according to several meta-analyses, including the treatment of anxiety and depression^20–22^ eating disorders^23^, posttraumatic stress^24^, or work-related stress^25^. Furthermore, EMH interventions find predominantly positive acceptance from both patients and mental health practitioners^26–28^. Thus, EMH interventions are viable and accepted tools in the treatment of mental disorders in outpatient settings.

Inpatient treatment signals an especially high need for timely and adequate intervention and is indicated for cases considered too severe for outpatient treatment^29^. Inpatient interventions can profit from supportive EMH procedures either to bridge waiting times, to blend with in-person interventions, or to ensure stabilization and relapse prevention in aftercare treatments. Especially the implementation of post-treatment aftercare improves the chances of a favorable and sustained development^30–32^. Thus, EMH treatment can be an important factor in the long-term success of inpatient treatments. Adequate aftercare enhances rehabilitation according to several reviews and meta-analyses^33–35^. Randomized controlled trials (RCT) exist on EMH treatment as add-ons to regular inpatient interventions^36^ and aftercare^37^. Furthermore, a systematic review^38^ found support for the efficacy of EMH aftercare treatments but was limited by the small number of studies. Yet as of now, there are to our knowledge no meta-analyses on the use of EMH in inpatient treatments, nor are there meta-analyses on EMH for inpatient aftercare. In addition, the systematic review on EMH inpatient care is several years old and does not incorporate the more recent research^38^.

The present study seeks to summarize the findings of previous RCTs on EMH treatments in inpatient settings in a meta-analysis. In addition, the risk of bias of the studies are assessed^39^. Specifically, this meta-analysis seeks to investigate 1) the total effect of EMH treatments on mental health outcomes in inpatient settings; 2) the effects of EMH treatments, divided into interventions and aftercare treatments; 3) the effect of EMH treatments in relationship depending on the mental health disorder; 4) the effect of type of therapy on EMH efficacy; 5) the effects of follow-up measures on EMH interventions are investigated to test long-term effects; and 6) the assessment of bias of the currently published RCT literature. Furthermore, post-hoc analyses were conducted to investigate 1) the role of EMH medium (e.g., app-based, web-based, SMS-based) on EMH treatment efficacy, and 2) the effect of the type of control group on EMH efficacy.

## Results

### Selected literature

A total of 26 research studies containing 123 effects and a sample size of *n* = 6112 (*intervention group* = 3041, *control group* = 3071) were included. A summary of the included studies is shown in Table 1. Five studies used blended treatment during inpatient stay while 21 studies conducted post-inpatient aftercare treatment. The most common patient groups (according to the number of studies) were eating disorders (*k* = 7) followed by mood disorders (*k* = 6), transdiagnostic (*k* = 4), psychotic disorders (*k* = 3), return to work treatments (*k* = 2), mental comorbidities with somatic disorders (*k* = 2), anxiety disorders (*k* = 1), and substance abuse (*k* = 1).

**Table 1:** Overview of studies

Study (authors, year, country)	Treatment type	Type of therapy	Patient group	Risk of Bias Assessment
Holländare et al. (2011), Sweden^65^	Aftercare	CBT	Mood disorder	Some concerns
Bauer et al. (2012), Germany^53^	Aftercare	CBT	Eating disorder	Low risk
Fichter et al. (2012), Germany^57^	Aftercare	CBT	Eating disorder	Some concerns
Bauer et al. (2013), Germany^54^	Aftercare	CBT	Eating disorder	Some concerns
Bischoff et al. (2013), Germany^72^	Aftercare	NA	Transdiagnostic	Low risk
Ebert et al. (2013a), Germany^58^	Aftercare	CBT	Transdiagnostic	Some concerns
Ebert et al. (2013b), Germany^73^	Aftercare	CBT	Transdiagnostic	High risk
Fichter et al. (2014), Germany^55^	Aftercare	CBT	Eating disorder	Some concerns
Gulec et al. (2014), Hungary^46^	Aftercare	NA	Eating disorder	Some concerns
Schmädeke et al. (2015), Germany^74^	Aftercare	CBT	Mood disorder	High risk
Harrington et al. (2016), USA^59^	Aftercare	NA	Substance abuse	Some concerns
Kordy et al. (2016), Germany^40^	Aftercare	NA	Mood disorder	Low risk
Willems et al. (2016), Netherlands^75^,	Intervention	CBT	Somatic comorbidity	High risk
Välimäki et al. (2017), Finland^50^	Aftercare	NA	Psychotic disorder	Low risk
Jacobi et al. (2017), Germany^41^	Aftercare	NA	Eating disorder	Some concerns
Zwerenz et al. (2017a), Germany^37^	Aftercare	PD	Transdiagnostic	Some concerns
Zwerenz et al. (2017b), Germany^76^	Aftercare	PD	Return to work	Some concerns
Zwerenz et al. (2017c), Germany^66^	Intervention	PD	Mood disorder	Some concerns
Norlund et al. (2018), Sweden^64^	Intervention	CBT	Somatic comorbidity	Low risk
Schlicker et al. (2018), Germany^77^	Aftercare	CBT	Mood disorder	High risk
Neumayr et al. (2019), Germany^56^	Aftercare	NA	Eating disorder	Some concerns
Zwerenz et al. (2019), Germany^78^	Aftercare	PD	Mood disorder	Some concerns
Alvarez et al. (2021), Australia^51^	Aftercare	NA	Psychotic disorder	Some concerns
Gallinat et al. (2021), Germany^42^	Aftercare	CBT	Psychotic disorder	Some concerns
Nolte et al. (2021), Germany^43^	Intervention	CBT	Mood disorder	Some concerns
Shaygan et al. (2021), Iran^79^	Intervention	NA	Somatic comorbidity	Low risk
Becker et al. (2022), Germany^45^	Aftercare	PD	Return to work	Some concerns
Levis et al. (2022), USA^80^	Intervention	Whole Health	Transdiagnostic	Low risk
Sharma et al. (2022), Canada^36^	Intervention	CBT	Anxiety symptoms	Some concerns
Bruhns et al. (2023), Germany^47^	Aftercare	CBT	Mood disorder	Some concerns

Studies included in risk of bias assessment (*k* = 30), categorized by treatment type, type of therapy, patient group, and assessed risk. Studies with a high risk were excluded for the meta analysis.

Thirteen out of 26 studies utilized a passive control group for which participants did not receive any type of active treatment (e.g., waiting list); eight studies used an active control group with an active treatment alternative to the EMH treatment (e.g., aftercare e-mail reminders for mental health tools, psychoeducation, rehabilitation activities, or psychosocial support such as counsling); five studies used an active control group to which the EMH treatment as added to in the intervention group; finally, one study used both active and passive control groups.

Three studies used SMS-based EMH interventions. Eighteen studies used web-based interventions such as SUMMIT^40^, IN@^41^, HEINS^42^, Deprexis^37,43,44^, GSA Online^45^, and EDINA^46^. Five studies used app-based tools such as MCT & More^47^ and Mindshift^36^. Each specified tool was used by only one study except for Deprexis, which was used in three studies.

Out of all included studies, 17 were conducted in Germany, two in Sweden and USA respectively, and one in Hungary, Iran, Finland, Canada, and Australia, respectively.

**Fig. 1:**
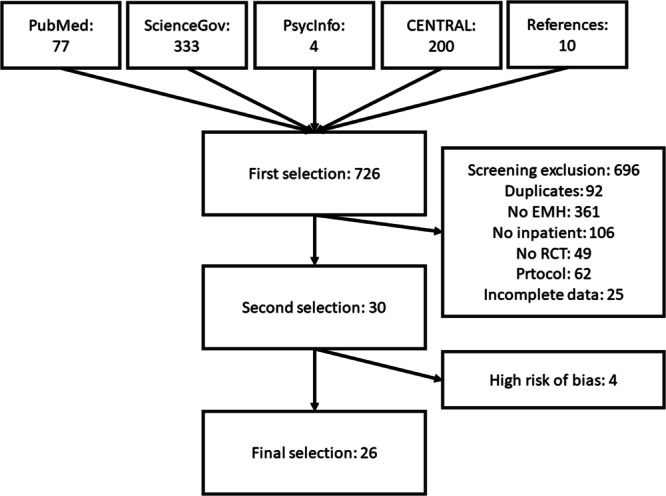
Study selection process. Flowchart depicting study selection. The first selection of 726 studies was found in five different databases. Following the evaluation by exclusion criteria, 30 studies were selected for risk of bias evaluation. After four studies were excluded for risk of bias, 26 studies were included in the meta-analysis. EMH e-mental health, RCT randomized controlled trial.

Study search and selection flow is depicted in Fig. 1.

### Risk of bias assessment

Four studies were rated as high risk of bias and excluded from the analysis. Out of the remaining studies, 19 were rated as medium risk of bias and seven as low risk of bias. Among the most common bias concerns were asymmetrical attrition rates in control and intervention groups, high attrition rates with unclear reasons, alternating allocations (rather than random allocation), and inadequate information on blinding procedures (e.g., no specifications for statements such as “the procedure was blinded”). All four high risk studies were excluded also due to unclear, high, or uneven attrition rates between groups.

The risk assessment is summarized in Table 1.

### Publication bias analyses

Preliminary analyses were conducted to test for publication bias using funnel plot and *p*-curve analyses.

#### Funnel plot analysis

**Fig. 2:**
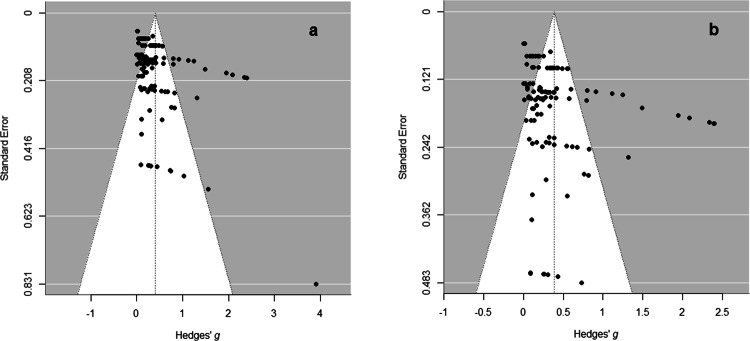
Funnel plots of included studies. Funnel plot across all effects (**a**) and after excluding studies with the largest standard errors (**b**). The funnel plots depict the effect sizes (Hedges’ *g*) plotted against the studies’ (reversed) standard errors. Asymmetry analyses found a significant asymmetry (**a**), but not when excluding four effects with the largest standard errors (**b**). As the effect size remains unaltered, the results do not indicate publication bias.

Funnel plots with effect sizes plotted against standard errors are depicted in Fig. 2a.

Publication bias would express itself in a preference for publishing significant compared to non-significant results. Because smaller studies need a higher effect size to reach significant effects compared to larger studies, an asymmetrical distribution with more smaller studies with larger effect sizes compared to larger studies would indicate publication bias. A regression analysis using standard error as a predictor of effect sizes suggests significant asymmetry (*z* = 3.6, *p* < 0.001, *i* = 123 effects).

Publication bias can be controlled by excluding the smallest studies^48^. After excluding studies with the largest standard errors (*i* = 4 effects, 3% of the total effects), another regression test showed no indicators of funnel plot asymmetry (z = 1.89, *p* = 0.058, *i* = 119, Fig. 2b). The total effect size remained unaltered (*g* = 0.33 [0.2, 0.46], *p* < 0.001), showing that the publication bias correction did not impact the results. Thus, the results do not indicate publication bias.

#### P-curve analysis

**Fig. 3:**
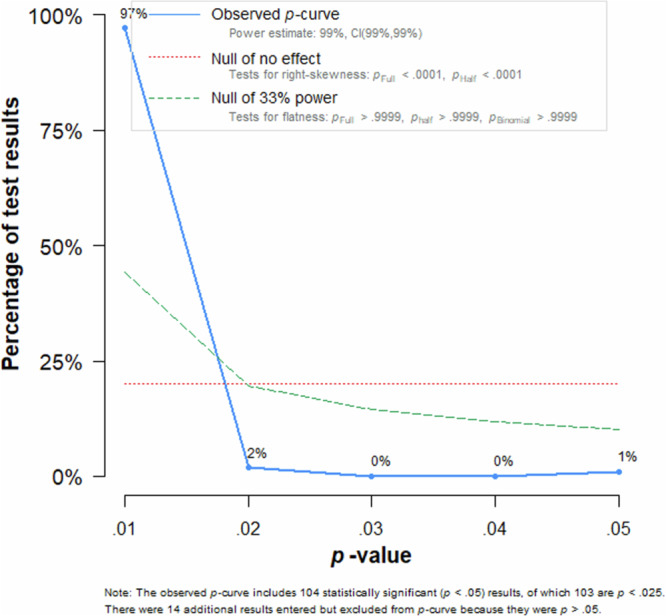
*P*-curve of included studies. *P*-curve including the meta-analysis’ 109 significant effects, compared to a hypothetical null-effect curve and a hypothetical 33% power effect curve. Analysis shows a significant right skewedness, indicating the existence of a true effect.

*P*-curve analysis was used to investigate publication bias further. A right-skewed *p*-curve would indicate an existing effect while a left-skewed *p*-curve would indicate publication bias or *p*-hacking as the latter curve would result from a tendency to acquire significant *p*-values of just below .05 despite the absence of a true effect indicated by a higher rate of results with smaller *p*-values. The *p*-curve is depicted in Fig. 3.

Out of all effects, *i* = 109 effects provided a significant effect size of *p* < 0.05, out of which *i* = 108 showed a *p*-value of *p* < 0.025. The significant right-skewedness test (*p*_binominal_ < 0.001, *z*_Full_ = -65.51, *z*_Half_ = -64.65, *p*_Half_ < 0.001) suggested the existence of a true effect. Furthermore, the non-significant flatness test (*p*_binominal_ = 1, *z*_Full_ = 64.13, *z*_Half_ = 65.88, *p*_Half_ = 1) provided no indicators that a true effect is not present.

In total, both funnel plot and *p*-curve analysis show no indicators of publication bias or *p*-hacking, and that the observed effect is true.

### Effect size analysis

**Fig. 4:**
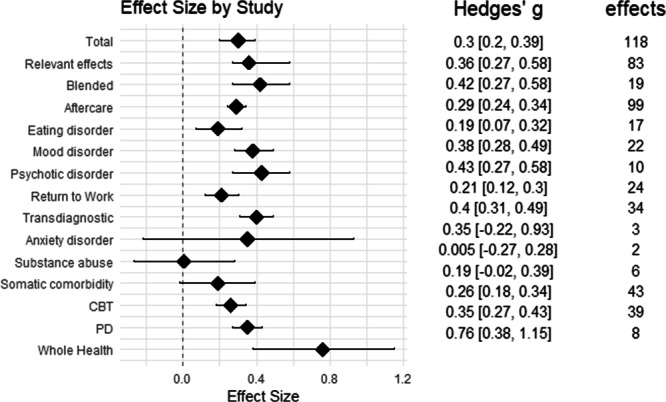
Synthesized effect sizes. Effect sizes, confidence intervals, and number of effects across conditions, controlled for study. Note. Total = across all data; relevant effects = only effects of measures relevant to the mental condition are included; blended = treatment with EMH blended with inpatient care; aftercare = treatment after inpatient care. CBT cognitive-behavioural therapy, PD Psychodynamic therapy.

A summary of all results is presented in Fig. 4.

#### Total effect

Total effect size with study as random effect revealed a significant positive effect of EMH intervention (*g* = 0.3 [0.2, 0.39], *p* < .001, *k* = 118). When only including effects of measures relevant to the mental disorder symptoms (e.g., Beck depression scores for depressive disorder patients) and removing measures not directly related to the mental disorder’s symptoms or clinical outcomes (e.g., social support, self-esteem), effect size increased (*g* = 0.36 [0.22, 0.5], *p* < 0.001, *k* = 83).

As expected given the variety of study designs and conditions, significant heterogeneity was observed for both the total effect (Q(117) = 408.25, *p* < .001) and when including only clinically relevant outcomes (Q(82) = 647.91, *p* < 0.001).

#### Treatment type

By-treatment type analysis revealed that both blended interventions during inpatient stay (*g* = 0.42 [0.27, 0.58], *p* < 0.001, *k* = 19) and aftercare treatments following inpatient stay (*g* = 0.29 [0.24, 0.34], *p* < 0.001, *k* = 99) showed significant effects.

#### Mental condition

By-condition analysis revealed significant effects of EMH interventions for eating disorder (*g* = 0.19 [0.07, 0.32], *p* = .003, *k* = 17), mood disorder (*g* = 0.38 [0.28, 0.49], *p* < 0.001, *k* = 22), psychotic disorder (*g* = 0.43 [0.27, 0.58], *p* < 0.001, *k* = 10), return to work (*g* = 0.21 [0.12, 0.3], *p* < 0.001, *k* = 24), and transdiagnostic patients (*g* = 0.4 [0.31, 0.49], *p* < 0.001, *k* = 34). No significant effects were found for anxiety disorders (*g* = 0.35 [−0.22, 0.93], *p* = 0.23, *k* = 3), mental comorbidity with somatic disorders (*g* = 0.19 [−0.02, 0.39], *p* = 0.072, *k* = 6), and substance abuse (*g* < 0.01 [−0.27, 0.28], *p* = 0.964, *k* = 2).

#### Type of therapy

Analysis by type of therapy revealed significant effects for cognitive behavioural therapy (CBT)-based treatments (*g* = 0.26 [0.18, 0.34], *p* < 0.001, *k* = 43) and psychodynamic (PD) treatments (*g* = 0.35 [0.27, 0.43], *p* < 0.001, *k* = 39).

#### Follow-up stability

To investigate potential effects of measurement time (e.g., a decrease of intervention efficacy for longer intervals after treatment), a linear mixed model with measurement time as the fixed effect and study as the random effect for effect sizes was calculated. Results showed no significant effect of measurement time (*t*(61) = −00.97, *p* = 0.337), showing no indication that the strength of the treatment effect is influenced by the time passed between intervention and measurement.

#### Post-hoc analyses

Post-hoc analyses were conducted to investigate differences between EMH medium/channel and effects of type of control group. EMH medium analysis revealed significant effects for EMH tools implemented as web-based tools (*g* = 0.32, CI [0.25, 0.37], *p* < 0.001) and multimedia interventions (*g* = 0.79, CI [0.29, 1.29], *p* = 0.002). Effects for app-based and SMS-based EMH tools were not significant. However, multimedia was used by only one study^43^. The only specific EMH tool used by multiple studies was Deprexis, which showed a significant effect (*g* = 0.61, CI [0.46, 0.77], *p* < 0.001).

Control group analysis revealed that EMH interventions significantly improve mental health outcomes compared to passive controls (no active treatment; *g* = 0.29, CI [0.19, 0.39], *p* < .001), active controls (active treatment alternative to EMH; *g* = 0.32, CI [0.24, 0.4], *p* < .001), and active controls to which the EMH intervention was added to in the intervention condition (*g* = 0.3, CI [0.22, 0.39], *p* < 0.001). Thus, EMH interventions show efficacy compared to active treatments and usual and when used in addition to usual treatments.

Few studies focused on patients affected by anxiety disorders, complicating interpretations of the presented results. Meanwhile, studies with transdiagnostic patients often included patients with anxiety disorders and measured anxiety symptoms (e.g., GAD-7). To gain further insight into the effects of EMH treatment on anxiety disoders, an additional post-hoc analysis has been conducted measuring the efficacy of EMH treatment on anxiety symptoms specifically. The analysis showed a significant effect on anxiety symptoms (*g* = 0.39, CI [0.18, 0.59], *p* < 0.001).

## Discussion

EMH procedures have shown to be a viable tool for the treatment of mental disorders, yet research on EMH in inpatient settings is relatively sparse. The current work presents, to our knowledge, the first meta-analysis providing evidence for the efficacy of EMH in inpatient treatment and aftercare. We found a significant small effect of EMH treatment (*g* = 0.3).When focusing on disorder symptoms and clinically relevant outcomes, the effect size is further increased (g = 0.36), signalling that EMH procedures are suitable as interventions tailored to mental disorders in inpatient settings. A preliminary analysis further found no indicators of publication bias or *p*-hacking within the literature.

The effect remained significant when dividing the studies into the common implementation types of EMH, first when blended with in-person inpatient treatment (*g* = 0.42) and second as an aftercare treatment following inpatient intervention (*g* = 0.29). The majority of studies (21 out of 26) used an aftercare setting with the goal to ensure stabilization and prevent relapse of inpatient cases. Inpatient cases tend to be more severe compared to outpatient cases, with worse post-treatment outcomes when not sufficiently supported by aftercare following discharge^30–32^. The present results suggest that EMH can provide such an effective tool, closing an important mental health supply gap.

By-disorder analysis found that EMH was especially effective for psychotic disorders (*g* = 0.42), transdiagnostic patient groups (*g* = 0.4), and mood disorders (*g* = 0.38). The results are comparable to meta-analyses finding small yet significant effects of EMH in outpatient settings for mood disorders^22^, providing evidence that the effects are comparable to inpatient settings.

The positive effect of EMH treatment for psychotic disorders is surprising given that EMH interventions may worsen psychotic patients’ concerns about technology and being recorded due to psychopathological paranoid tendencies^49^. Furthermore, the effect contrasts the negative outcomes reported in studies investigating psychotic patients^42,50,51^. While the results complement previous research on the effectiveness of EMH outpatient treatments for schizophrenia and psychosis^52^, the usage of EMH interventions for psychotic disorders remains not well developed, and their efficacy cannot be reliably estimated with the current research. For inpatient settings, SMS-based aftercare reminders for medication adherence did not improve patient outcomes^50^. The HEINS web-based aftercare program containing multiple modules (including psychoeducation, crisis plans, contacts to psychiatrists, and supportive monitoring) meanwhile showed positive user acceptance and adherence^42^, and Horyzons, an online social therapy aftercare program containing multiple features (including psychoeducation, skill development support, peer-to-peer conversations, and expert support), improved patient employment and reduced emergency room visits compared to usual care^51^. Given that both Horyzons and HEINS are interactive support units containing multiple modules, the results suggest that more extensive EMH treatment is needed to ensure aftercare of patients with psychosis. Patients with severe illnesses such as psychosis may not be able to effectively utilize digital health tools. EMH tools are to be used with caution when treating patients with psychosis and should be used in addition to in-person treatment instead of an alternative.

For outpatient treatment, the efficacy of EMH treatment for anorexia nervosa is not well researched, potentially due to the severity of the disorder and the presumed necessity for face-to-face treatment by clinicians^21^. Out of seven studies investigating eating disorder patients, four focused mainly on bulimia nervosa^41,46,53,54^. When excluding a follow-up study^55^ and a pilot RCT^56^, only one proper RCT study focused on anorexia nervosa^57^. Although the initial results are promising, caution should be taken when transferring the results onto patients with anorexia nervosa given that the disorder leads to severe consequences including somatic complications that may be insufficiently tracked and treated through digital means.

Meanwhile, no significant effects for anxiety symptoms, comorbidity with somatic disorders, or substance abuse disorders were found. However, only one study investigated anxiety symptoms^36^. Meanwhile, multiple studies with transdiagnostic patient groups included patients with anxiety disorders^37^^,39^^,58^. A post-hoc analysis focusing on anxiety symptoms revealed a significant effect (*g* = 0.39). Inpatient treatment is typically not indicated for anxiety disorders, which may explain the low number of studies. Given that EMH interventions are effective in treating anxiety disorders in outpatient settings^22^, and that the post-hoc analysis revealed a significant improvement in anxiety symptoms, the current negative findings on EMH inpatient treatment for anxiety disorders are to be interpreted with caution. A similar caution can be expressed for the negative result on substance abuse patients, which has been investigated by only one study^59^. Furthermore, future research ought to differentiate effects of EMH for different anxiety diagnoses in inpatient care, as EMH outpatient treatment effectiveness has been found to differ across anxiety disorders^22^.

Analysis by type of therapy revealed the effectiveness of both CBT- (*g* = 0.26) and PD- (*g* = 0.35) based interventions, showing that EMH treatment is effective when based on either of these types of psychotherapy

Finally, the result that observation period did not affect outcomes indicates that EMH-based treatment effects do not deteriorate with time passed after treatment, indicating the long-term stability of the effects. However, the latest measurement used in this analysis was 24 months after treatment. Hence, results cannot be interpreted for longer periods.

In general, the meta-analysis shows the efficacy of EMH treatment across different mental health disorders and types of therapy. Hence, mental health treatment can profit from integrating EMH into the patient journey. Given that EMH add-on also significantly improves outcomes compared to a regular active control group (*g* = 0.3), adding EMH to regular practices can improve overall treatment outcomes. Since treatment as usual tends to be minimal for aftercare treatment, EMH can facilitate long-term improvements and remission prevention following inpatient treatment since other aftercare practices are lacking or minimal. Especially web-based EMH treatment has been shown to be effective throughout multiple studies (*g* = 0.32) compared to SMS- or app-based approaches. Hence, practitioners may use EMH tools both as additives and as alternatives to regular treatment, and especially for aftercare following inpatient treatment. Web-based EMH tools have shown efficacy in most studies.

The meta-analysis is limited by the small number of studies especially for subgroup analyses, as some subgroups (e.g., anxiety disorder or substance abuse patients, or whole health approaches) only include a single study each and can thus not be properly interpreted. Although a total effect was found with a sufficient number of trials, further RCT research is needed to conduct more conclusive meta-analyses for subgroup-related research areas.

The small number of studies precludes further interesting analyses relevant to the design and implementation of EMH methods. For example, a previous meta-analysis on outpatient settings found that specific EMH methods were more effective for certain disorders (e.g., chatbots for depression, mood monitoring features for anxiety). Such research questions may be tackled in future meta-analyses when an adequate number of RCTs have been conducted. Meta-analyses and reviews are generally limited by the terms used and search outputs when conducting literature searches. Even though two literature searches (February 2024 and July 2024) were done for this meta-analysis, it may still not include all relevant literature. Furthermore, this meta-analysis was not preregistered. However, all relevant documents are publicly available.

Specific neuropsychological and cognitive measures were excluded from this meta-analysis to focus the research on explicit mental health outcomes. However, mental health deficits often co-occur with cognitive deficits, for example in memory, concentration, or problem-solving tasks. Although disorder-specific questionnaire measures encompass the measurement of such deficits, future research can focus on the effect of EMH interventions for the improvement of cognitive skills in patients affected by mental health disorders specifically.

Out of all 26 included studies 20 were conducted in Western or Northern Europe (17 in Germany, two in Sweden, one in Finland), three were conducted in North America (two in the USA and one in Canada), one in Australia, one in Hungary, and one in Iran. Research from other regions, such as Africa or East Asia, was absent. This may be due to differences in healthcare systems in different regions, and treatments alternative to inpatient treatment for more severe health cases. Thus, the results of this meta-analysis are mainly derived from studies conducted in countries with populations majorly of European descent. In order to generalize the reported findings, future research may aim to investigate EMH tools in more diverse populations.

Engagement and adherence are major concerns when applying EMH tools^60–63^. Effects of EMH on attrition were mitigated in this analysis by including group attrition effects in the RoB assessment: in fact, all four high risk studies were excluded due to unclear or uneven attrition rates. Engagement can be defined as usage as intended, measured for example through use frequency or completion^60^. Various included studies excluded participants with low engagement despite completion^41^ and hence controlled for low engagement. Included studies mostly did not report direct effects on engagement on outcomes. One study found no effect of EMH tool use (assessed via logs) on symptom severity^56^. Similarly, other studies did not find a correlation between EMH use frequency and symptom improvement^47^, completed models and symptom improvement^64^, or differences between high- and low-frequency users^59^. Meanwhile, the number of completed EMH courses did significantly improve symptoms in patients with anorexia nervosa^55^. Although there are only few studies and results are not consistent, the results nevertheless indicate that use frequency or intensity does generally not affect the treatment efficacy. Finally, some studies report improved engagement in the intervention compared to a control group^65,66^, indicating that EMH intervention may improve engagement behaviour. Future research may investigate effects of such engagement when implementing EMH tools.

Given that various measurement outcomes were used and summarized to generalize a wider range of findings, results do not consistently reflect the most clinically relevant outcomes (e.g., remission or relapse rates) which were only reported by six studies for varying mental disorders. Instead, the majority of research studies relied on symptom questionnaires. In total, a majority of the studies included were assessed with some concerns regarding risk of bias. Due to the low number of high-quality research with low bias and large sample sizes, results should be interpreted with some degree of caution. EMH implementations furthermore involve certain risks^67^ such as a lack of quality standards^68^, data privacy issues^18^, or a lack of digital literacy by practitioners^19^. Despite promising results in this meta analysis, in the context of such risks, more high quality RCT research is necessary for a more rigorous assessment of EMH efficacy.

In conclusion, the results indicate that EMH procedures are an effective tool in the treatment and aftercare of inpatients, especially for psychotic, mood disorder, and eating disorder, and patient groups combining different diagnoses. EMH tools can be used both in addition to in-person treatment and when in-person treatment is not available, e.g., for aftercare. Future research should investigate effects of EMH tools for the inpatient treatment of specific disorders and the relevance of the specific tools used. Larger sample sizes and randomized trials are warranted to substantiate these effects.

## Methods

This review was conducted in accordance to the Preferred Reporting Items for Systematic Reviews and Meta-Analyses (PRISMA) guidelines^69^ and Cochrane Handbook guidelines for meta-analyses and systematic reviews^39^.

### Literature

#### Literature search

The literature databases SciencGov, PsycInfo, PubMed, and CENTRAL were searched for published literature. In addition, the ProQuest Database was searched for dissertation theses, and ICTRP and ClinicalTrials were searched for trial result registers.

To aim for high sensitivity according to Cochrane guidelines^39^, we used multiple search terms in relation to the following topics: e-mental health (digital, online, e-mental health, technology-based, web-based, internet-based, mobile-based), treatment setting (psychotherapy, psychiatric, psychosomatic), inpatient setting (inpatient, ward patient, hospitalized), and experimental design (RCT, randomized controlled trial). The search term used corresponds to the following: (“digital” OR “online” OR “e-mental health” OR “technology-based” OR “web-based” OR “internet-based” OR “mobile-based”) AND (“psychotherapy” OR “psychiatric” OR “psychosomatic”) AND (“inpatient” OR “ward patient” OR “hospitalized”) AND (“RCT” OR “randomized controlled trial”). Two researchers conducted the literature search in February 2024. Literature search was performed in English and German.

A secondary search was conducted in July 2024 by extending the search to use the terms “e-health”, “mhealth”, and “telemedicine”, and using the mesh terms “digital health”, “telemedicine”, “psychotherapy”, “psychosomatic medicine”, and “inpatients” if applicable, for the databases CENTAL and PubMed. The secondary literature search did not yield any new viable studies.

#### Literature selection

We included research studies providing EMH interventions during inpatient treatment or aftercare following inpatient treatment, and studies investigating psychiatric symptoms co-occurring in patients hospitalized for physical conditions (e.g., stress or depression symptoms in cancer patients). Cluster and pilot RCTs were included as well.

Studies were excluded if they 1) did not investigate the effect of EMH intervention or aftercare methods, 2) did not investigate inpatients (either during or after inpatient intervention), 3) did not investigate mental health measures as treatment outcomes (e.g., only focusing on somatic symptoms or acceptability of the intervention; specific neuropsychological or cognitive outcomes like problem-solving skills were also excluded), 4) were not randomized controlled trials, 5) did not provide sufficient information to extract the relevant data (e.g., outcome measures or sample sizes), and 6) showed a high risk of bias assessed via the Risk of Bias tool (see next section)^39^. Neuropsychological or cognitive were excluded to focus the meta-analysis on mental health treatment effects. Although cognitive or neuropsychological deficits can be symptoms of mental health disorders, symptom-focused measures of mental health deficits (e.g., depressiveness questionnaires for clinical depression) provide a more discriminative estimation of mental health deficits.

Three independent raters took part in the literature selection. In case of disagreements, the raters discussed the study until agreement was found.

#### Risk of bias assessment

Risk of bias was assessed using the Cochrane risk-of-bias tool for randomized trials (RoB 2)^39^. RoB 2 is designed to assess the risk of an RCT’s bias by classifying the level of risk for the following domains: random sequence generation, allocation concealment, blinding of participants and personnel, blinding of outcome assessment, incomplete outcome data, and selective reporting. Examples of risk of bias include non-random or semi-random participant grouping (incl. alternating allocation); high, uneven, or unexplained participant attrition between groups; lack of blinding; or unreported discrepancies between the study protocol and study. If no information on a domain was provided, the particular domain was assessed with medium risk.

Domains were rated on three levels: low, medium, or high risk of bias. Research studies with a high risk of bias were excluded from the analysis.

### Measurement selection

For the total analyses, only measures related to clinical symptoms and psychosocial performance were included. These include: metric variables of disorder-related incidents (relapses, readmissions, abstinence, admissions); disorder-related symptom severity measurements; general psychopathology, well-being, or quality of life; employment-related measures (when relevant); and specific mental or psychosocial measures expected to correlate with symptom severity (e.g., self-esteem, positive and negative affect, stress). All relevant measures in a study were included in an analysis and controlled by treating study as a random effect.

### Variable summarization

To investigate the relevant research questions, studies and measures were categorized by the following system.

*EMH treatment type* was categorized into either *blended intervention* (EMH was implemented into the inpatient setting) or *aftercare treatment* (EMH was provided after completing inpatient setting).

The variable *Disorder type* was classified into the following categories based on the patient group investigated in the study: *anxiety disorders* (ICD-10 diagnoses F40 and F41), *eating disorders* (ICD-10 diagnoses F50) *mood disorders* (ICD-10 diagnoses F3), *psychotic disorders* (ICD-10 diagnoses F2), *substance abuse disorders* (ICD-10 diagnoses F1x.2), or their DSM-5 diagnostic equivalents. A study treating patient groups from different categories was classified as *transdiagnostic*. A study was categorized as *somatic comorbidity* if the effects of EMH interventions on mental health outcomes in somatic inpatient groups were investigated (e.g., stress or anxiety symptoms in cancer patients). Finally, the category *return to work* was used for studies focussing on outcomes related to workplace reintegration following inpatient care.

The variable *type of therapy* was classified according to the type of therapy the EMH intervention was based on according to the authors. If no type of therapy was mentioned, the variable was valued as *not available*.

### Data extraction

Data was summarized on multiple variables: author, title, year, country, type (aftercare, blended treatment), treated mental disorder, somatic illness (if present), digital method, type of therapy, type of control group (active, passive), outcome measure, follow-up, sample sizes, and outcome results (means, standard deviations, odds ratios, effect sizes). Data was extracted by one rater and verified by two other independent raters. Study characteristics were tabulated according to the planned subgroup analyses. Studies with insufficient data were excluded from the (sub-)analyses.

### Data transformation

Hedges’ *g* was used to report effect sizes as it outperforms Cohen’s *d* for small sample sizes. Cohen’s *d* effect sizes and variances were transformed to Hedges’ *g* values and variances using the following formulas^48^:

1 $$g=d\,\left(1-\frac{3}{4{df}-1}\right)$$

2 $${vg}={vd}\,\left(1-\frac{3}{4{df}-1}\right)2$$

When a study reported odds ratio (OR) values, values were first transformed into Cohen’s *d* using the following formula^48^:

3 $$d=\mathrm{log}\left({OR}\right)\frac{\sqrt{3}}{\,\pi }$$

Cohen’s *d* values were then transformed into Hedges’ *g* according to Formula 1.

### Data analysis

Heterogeneity was tested and pre-assumed given the variety of setups in research studies and subgroup analyses were therefore decided a priori. Fixed-random effects models were used with study as a random factor. To assess the results’ robustness, the total effect is analysed two times, first using the whole range of data, and second using only outcomes that are clinically relevant (limited to symptom severity and clinical outcomes). Effects’ certainty and confidence were assessed through risk of bias assessment according to Cochrane guidelines and by investigating publication bias using funnel plot and *p*-curve analyses. The meta-analysis was not preregistered. No protocol is available for the meta-analysis. Confidence was assessed by calculating confidence intervals from standard errors.

Post-hoc analyses were decided after the data was analysed for the main hypotheses. Post-hoc analyses included the effect of EMH medium, the role of control group, and the effect of EMH on anxiety symptoms specifically.

## Supplementary information

Supplementary Information

## Data Availability

Data including the complete list of searched literature, the included studies, extracted data, and risk assessment are publicly available at https://osf.io/bc59e. Thus, all data is provided to replicate assessment of literature according to inclusion criteria and risk of bias, as well as all data necessary to replicate the analyses.

## Code availability

The R code for the analysis is publicly available at https://osf.io/bc59e. Main and subgroup analyses and visualization of results were conducted via RStudio (ver. 2021.9.1.0, R version 4.1.2). The R packages dmetar and metafor were used for the analyses^70,71^.

## References

[1] Borges, G. et al. Twelve-month prevalence of and risk factors for suicide attempts in the World Health Organization World Mental Health Surveys. *J. Clin. Psychiatry* **71**, 1617–1628 (2010). 20816034 10.4088/JCP.08m04967blu PMC3000886

[2] Doran, C. M. & Kinchin, I. A review of the economic impact of mental illness. *Aust. Health Rev.* **43**, 43 (2019). 29129189 10.1071/AH16115

[3] Vos, T. et al. Global, regional, and national incidence, prevalence, and years lived with disability for 328 diseases and injuries for 195 countries, 1990–2016: A systematic analysis for the global burden of disease study 2016. *Lancet* **390**, 1211–1259 (2017). 28919117 10.1016/S0140-6736(17)32154-2 PMC5605509

[4] Santomauro, D. F. et al. Global prevalence and burden of depressive and anxiety disorders in 204 countries and territories in 2020 due to the COVID-19 pandemic. *Lancet* **398**, 1700–1712 (2021). 34634250 10.1016/S0140-6736(21)02143-7 PMC8500697

[5] Ahmed, N. et al. Mental health in Europe during the COVID-19 pandemic: A systematic review. *Lancet Psychiatry* **10**, 537–556 (2023). 37321240 10.1016/S2215-0366(23)00113-X PMC10259832

[6] Alonso, J. et al. Prevalence of mental disorders in Europe: Results from the European study of the epidemiology of Mental Disorders (esemed) project. *Acta Psychiatr. Scandinavica* **109**, 21–27 (2004). 10.1111/j.1600-0047.2004.00327.x 15128384

[7] Sacco, R., Camilleri, N., Eberhardt, J., Umla-Runge, K. & Newbury-Birch, D. A systematic review and meta-analysis on the prevalence of mental disorders among children and adolescents in Europe. European Child & Adolescent Psychiatry 10.1007/s00787-022-02131-2 (2022). 10.1007/s00787-022-02131-2 PMC9800241 36581685

[8] Wittchen, H. U. et al. The size and burden of mental disorders and other disorders of the brain in Europe 2010. *Eur. Neuropsychopharmacol.* **21**, 655–679 (2011). 21896369 10.1016/j.euroneuro.2011.07.018

[9] Zuberi, A. et al. Prevalence of mental disorders in the WHO Eastern Mediterranean Region: A systematic review and meta-analysis. *Front. Psychiatry* **12**, (2021). 10.3389/fpsyt.2021.665019 PMC8316754 34335323

[10] Jacobi, F. et al. Psychische Störungen in der Allgemeinbevölkerung. *Der Nervenarzt* **85**, 77–87 (2014). 24441882 10.1007/s00115-013-3961-y

[11] Andrade, L. H. et al. Barriers to mental health treatment: Results from the WHO World Mental Health Surveys. *Psychol. Med.* **44**, 1303–1317 (2013). 23931656 10.1017/S0033291713001943 PMC4100460

[12] EU-Compass for action on Mental Health and well-being. Public Health Available at: https://health.ec.europa.eu/non-communicable-diseases/mental-health/eu-compass-action-mental-health-and-well-being_en (Accessed: 21st May 2024).

[13] Koopmanschap, M. A., Brouwer, W. B. F., Hakkaart-van Roijen, L. & van Exel, N. J. A. Influence of waiting time on cost-effectiveness. *Soc. Sci. Med.* **60**, 2501–2504 (2005). 15814175 10.1016/j.socscimed.2004.11.022

[14] Reichert, A. & Jacobs, R. The impact of waiting time on patient outcomes: Evidence from early intervention in psychosis services in England. *Health Econ.* **27**, 1772–1787 (2018). 30014544 10.1002/hec.3800 PMC6221005

[15] van Dijk, D. A. et al. Worse off by waiting for treatment? the impact of waiting time on clinical course and treatment outcome for depression in routine care. *J. Affect. Disord.* **322**, 205–211 (2023). 36372129 10.1016/j.jad.2022.11.011

[16] Sherman, M. L., Barnum, D. D., Buhman-Wiggs, A. & Nyberg, E. Clinical intake of child and adolescent consumers in a rural community mental health center: Does wait-time predict attendance? *Community Ment. Health J.* **45**, 78–84 (2008). 18807182 10.1007/s10597-008-9153-8

[17] Williams, M. E., Latta, J. & Conversano, P. Eliminating the wait for Mental Health Services. *J. Behav. Health Serv. Res.* **35**, 107–114 (2007). 17975730 10.1007/s11414-007-9091-1

[18] Köhnen, M., Dirmaier, J. & Härter, M. Potenziale und Herausforderungen von E-mental-health-interventionen in der Versorgung Psychischer störungen. *Fortschr. der Neurologie · Psychiatr.* **87**, 160–164 (2019). 10.1055/a-0853-2568 30891717

[19] Weitzel, E. C. et al. E-mental health in Germany — what is the current use and what are experiences of different types of health care providers for patients with mental illnesses? *Arch. Public Health* **81**, 1–6 (2023). 37461064 10.1186/s13690-023-01150-y PMC10353209

[20] Bolinski, F. et al. The effect of E-mental health interventions on academic performance in university and college students: A meta-analysis of randomized controlled trials. *Internet Interventions* **20**, 100321 (2020). 32382515 10.1016/j.invent.2020.100321 PMC7201188

[21] Firth, J. et al. Can smartphone mental health interventions reduce symptoms of anxiety? A meta-analysis of randomized controlled trials. *J. Affect. Disord.* **218**, 15–22 (2017). 28456072 10.1016/j.jad.2017.04.046

[22] Linardon, J. et al. Current evidence on the efficacy of mental health smartphone apps for symptoms of depression and anxiety. A meta‐analysis of 176 randomized controlled trials. *World Psychiatry* **23**, 139–149 (2024). 38214614 10.1002/wps.21183 PMC10785982

[23] Linardon, J., Shatte, A., Messer, M., Firth, J. & Fuller-Tyszkiewicz, M. E-mental health interventions for the treatment and prevention of eating disorders: An updated systematic review and meta-analysis. *J. Consulting Clin. Psychol.* **88**, 994–1007 (2020). 10.1037/ccp0000575 32852971

[24] Simblett, S., Birch, J., Matcham, F., Yaguez, L. & Morris, R. A systematic review and meta-analysis of E-mental health interventions to treat symptoms of posttraumatic stress. JMIR Mental Health **4**, (2017). 10.2196/mental.5558 PMC5451639 28526672

[25] Stratton, E. et al. Effectiveness of ehealth interventions for reducing mental health conditions in employees: A systematic review and meta-analysis. PLOS ONE **12**, (2017). 10.1371/journal.pone.0189904 PMC5739441 29267334

[26] Löbner, M. et al. What comes after the trial? an observational study of the real-world uptake of an e-mental health intervention by general practitioners to reduce depressive symptoms in their patients. *Int. J. Environ. Res. Public Health* **19**, 6203 (2022). 35627739 10.3390/ijerph19106203 PMC9142114

[27] Rost, T. et al. User acceptance of computerized cognitive behavioral therapy for depression: Systematic review. Journal of Medical Internet Research **19**, (2017). 10.2196/jmir.7662 PMC5617907 28903893

[28] Wangler, J. & Jansky, M. How can primary care benefit from Digital Health Applications? – a quantitative, explorative survey on attitudes and experiences of General Practitioners in Germany. *BMC Digital Health* **2**, 1–14 (2024).

[29] Zipfel, S., Herzog, W., Kruse, J. & Henningsen, P. Psychosomatic medicine in Germany: More timely than ever. *Psychother. Psychosom.* **85**, 262–269 (2016). 27509065 10.1159/000447701

[30] Franz, M. et al. Stationäre Tiefenpsychologisch Orientierte psychotherapie bei Depressiven Störungen (stop-D) - erste befunde einer naturalistischen, multizentrischen Wirksamkeitsstudie. *Z. f.ür. Psychosomatische Med. und Psychotherapie* **61**, 19–35 (2015). 10.13109/zptm.2015.61.1.19 25831982

[31] Gönner, S., Bischoff, C., Ehrhardt, M. & Limbacher, K. Effekte therapiezielorientierter Kognitiv-Verhaltenstherapeutischer Nachsorgemaßnahmen auf den therapietransfer im Anschluss an eine Stationäre psychosomatische Rehabilitationsbehandlung. *Die Rehabilitation* **45**, 369–376 (2006). 17123219 10.1055/s-2006-932614

[32] Zeeck, A., Wietersheim, Jvon, Weiss, H., Beutel, M. & Hartmann, A. The INDDEP Study: Inpatient and day hospital treatment for depression – symptom course and predictors of change. *BMC Psychiatry* **13**, 100 (2013). 23531019 10.1186/1471-244X-13-100 PMC3616996

[33] Giel, K. E. et al. Efficacy of post-inpatient aftercare treatments for anorexia nervosa: A systematic review of randomized controlled trials. *J. Eat. Disord.* **9**, 129 (2021). 34654471 10.1186/s40337-021-00487-5 PMC8518230

[34] Hegedüs, A., Kozel, B., Richter, D. & Behrens, J. Effectiveness of transitional interventions in improving patient outcomes and service use after discharge from psychiatric inpatient care: A systematic review and meta-analysis. *Front. Psychiatry* **10**, 969 (2020). 32038320 10.3389/fpsyt.2019.00969 PMC6985781

[35] Vittengl, J. R., Clark, L. A., Dunn, T. W. & Jarrett, R. B. Reducing relapse and recurrence in Unipolar Depression: A comparative meta-analysis of cognitive-behavioral therapy’s effects. *J. Consulting Clin. Psychol.* **75**, 475–488 (2007). 10.1037/0022-006X.75.3.475 PMC2630051 17563164

[36] Sharma, G. et al. Brief app-based cognitive behavioral therapy for anxiety symptoms in psychiatric inpatients: Feasibility randomized controlled trial. *JMIR Formative Res.* **6**, e38460 (2022). 10.2196/38460 PMC9669882 36322113

[37] Zwerenz, R. et al. Transdiagnostic, psychodynamic web-based self-help intervention following inpatient psychotherapy: Results of a feasibility study and randomized controlled trial. *JMIR Ment. Health* **4**, e41 (2017). 29038094 10.2196/mental.7889 PMC5662790

[38] Hennemann, S., Farnsteiner, S. & Sander, L. Internet- and Mobile-based aftercare and relapse prevention in mental disorders: A systematic review and recommendations for Future Research. *Internet Interventions* **14**, 1–17 (2018). 30510909 10.1016/j.invent.2018.09.001 PMC6205252

[39] Cochrane Handbook for Systematic Reviews of interventions. (Cochrane, 2019).

[40] Kordy, H. et al. Internet-delivered disease management for recurrent depression: A multicenter randomized controlled trial. *Psychother. Psychosom.* **85**, 91–98 (2016). 26808817 10.1159/000441951

[41] Jacobi, C. et al. Web-based aftercare for women with bulimia nervosa following inpatient treatment: Randomized controlled efficacy trial. *J. Med. Internet Res.* **19**, e321 (2017). 28939544 10.2196/jmir.7668 PMC5630693

[42] Gallinat, C. et al. Feasibility of an intervention delivered via mobile phone and internet to improve the continuity of care in schizophrenia: A randomized controlled pilot study. *Int. J. Environ. Res. Public Health* **18**, 12391 (2021). 34886117 10.3390/ijerph182312391 PMC8656751

[43] Nolte, S. et al. Do sociodemographic variables moderate effects of an internet intervention for mild to moderate depressive symptoms? an exploratory analysis of a randomised controlled trial (evident) including 1013 participants. *BMJ Open* **11**, e041389 (2021). 33500282 10.1136/bmjopen-2020-041389 PMC7839881

[44] Abadi, M. et al. Achieving whole health: A preliminary study of TCMLH, a group-based program promoting self-care and empowerment among veterans. *Health Educ. Behav.* **49**, 347–357 (2021). 34018443 10.1177/10901981211011043

[45] Becker, J., Kreis, A., Beutel, M. E. & Zwerenz, R. Wirksamkeit der Internetbasierten, Berufsbezogenen Nachsorge GSA-online im Anschluss an die stationäre psychosomatische Rehabilitation: Ergebnisse einer randomisiert Kontrollierten Studie. *Die Rehabilitation* **61**, 276–286 (2022). 35995057 10.1055/a-1871-4484

[46] Gulec, H. et al. A randomized controlled trial of an internet-based posttreatment care for patients with eating disorders. *Telemed. e-Health* **20**, 916–922 (2014). 10.1089/tmj.2013.0353 25188398

[47] Bruhns, A., Lüdtke, T., Moritz, S. & Bücker, L. A mobile-based intervention to increase self-esteem in students with depressive symptoms: Randomized controlled trial. *JMIR mHealth uHealth* **9**, e26498 (2021). 34255711 10.2196/26498 PMC8314153

[48] Borenstein, M. Computing effect sizes for meta-analysis. (Wiley-Blackwell, 2011).

[49] Philippe, T. J. et al. Digital Health Interventions for delivery of mental health care: Systematic and comprehensive meta-review. *JMIR Ment. Health* **9**, e35159 (2022). 35551058 10.2196/35159 PMC9109782

[50] Välimäki, M., Kannisto, K. A., Vahlberg, T., Hätönen, H. & Adams, C. E. Short text messages to encourage adherence to medication and follow-up for people with psychosis (mobile.net): Randomized controlled trial in Finland. *J. Med. Internet Res.* **19**, e245 (2017). 28701292 10.2196/jmir.7028 PMC5529737

[51] Alvarez‐Jimenez, M. et al. The Horyzons Project: A randomized controlled trial of a novel online social therapy to maintain treatment effects from specialist first‐episode psychosis services. *World Psychiatry* **20**, 233–243 (2021). 34002511 10.1002/wps.20858 PMC8129860

[52] Clarke, S., Hanna, D., Mulholland, C., Shannon, C. & Urquhart, C. A systematic review and meta-analysis of digital health technologies effects on psychotic symptoms in adults with psychosis. *Psychosis* **11**, 362–373 (2019).

[53] Bauer, S., Okon, E., Meermann, R. & Kordy, H. Technology-enhanced maintenance of treatment gains in eating disorders: Efficacy of an intervention delivered via text messaging. *J. Consulting Clin. Psychol.* **80**, 700–706 (2012). 10.1037/a0028030 22545736

[54] Bauer, S., Okon, E., Meermann, R. & Kordy, H. SMS-Nachsorge: Sektorenübergreifende Versorgung für patientinnen mit bulimia nervosa. *Verhaltenstherapie* **23**, 204–209 (2013).

[55] Fichter, M. M., Quadflieg, N. & Lindner, S. Internet-based relapse prevention for anorexia nervosa: Nine- month follow-up. *J. Eat. Disord.* **1**, 23 (2013). 24999404 10.1186/2050-2974-1-23 PMC4081799

[56] Neumayr, C., Voderholzer, U., Tregarthen, J. & Schlegl, S. Improving aftercare with technology for anorexia nervosa after intensive inpatient treatment: A pilot randomized controlled trial with a therapist‐guided smartphone app. *Int. J. Eat. Disord.* **52**, 1191–1201 (2019). 31429974 10.1002/eat.23152

[57] Fichter, M. M. et al. Does internet-based prevention reduce the risk of relapse for anorexia nervosa? *Behav. Res. Ther.* **50**, 180–190 (2012). 22317754 10.1016/j.brat.2011.12.003

[58] Ebert, D., Tarnowski, T., Gollwitzer, M., Sieland, B. & Berking, M. A transdiagnostic internet-based maintenance treatment enhances the stability of outcome after inpatient cognitive behavioral therapy: A randomized controlled trial. *Psychother. Psychosom.* **82**, 246–256 (2013). 23736751 10.1159/000345967

[59] Harrington, K. F. et al. Web-based smoking cessation intervention that transitions from inpatient to outpatient: Study protocol for a randomized controlled trial. *Trials* **13**, 123 (2012). 22852802 10.1186/1745-6215-13-123 PMC3533743

[60] Kernebeck, S., Busse, T. S., Ehlers, J. P. & Vollmar, H. C. Adhärenz digitaler Interventionen im Gesundheitswesen: Definitionen, Methoden und offene Fragen [Adherence to digital health interventions: definitions, methods, and open questions]. *Bundesgesundheitsblatt Gesundheitsforschung Gesundheitsschutz* **64**, 1278–1284 (2021). OctGermanEpub 2021 Sep 24. PMID: 34559252; PMCID: PMC8492574. 34559252 10.1007/s00103-021-03415-9 PMC8492574

[61] Sieverink, F., Kelders, S. M. & van Gemert-Pijnen, J. E. Clarifying the Concept of Adherence to eHealth Technology: Systematic Review on When Usage Becomes Adherence. *J. Med Internet Res* **19**, e402 (2017). Dec 6PMID: 29212630; PMCID: PMC5738543. 29212630 10.2196/jmir.8578 PMC5738543

[62] Beatty, L. & Binnion, C. A Systematic Review of Predictors of, and Reasons for, Adherence to Online Psychological Interventions. *Int J. Behav. Med* **23**, 776–794 (2016). DecPMID: 26957109. 26957109 10.1007/s12529-016-9556-9

[63] Eysenbach, G. The law of attrition. *J. Med. Internet Res.* **7**, e11 (2005). Mar 31PMID: 15829473; PMCID: PMC1550631. 15829473 10.2196/jmir.7.1.e11 PMC1550631

[64] Norlund, F. et al. Internet-based cognitive behavioral therapy for symptoms of depression and anxiety among patients with a recent myocardial infarction: The U-CARE heart randomized controlled trial. *J. Med. Internet Res.* **20**, e88 (2018). 29519777 10.2196/jmir.9710 PMC5874001

[65] Holländare, F. et al. Randomized trial of internet-based relapse prevention for partially remitted depression. *Acta Psychiatr. Scandinavica* **124**, 285–294 (2011). 10.1111/j.1600-0447.2011.01698.x 21401534

[66] Zwerenz, R. et al. Online self-help as an add-on to inpatient psychotherapy: Efficacy of a new blended treatment approach. *Psychother. Psychosom.* **86**, 341–350 (2017). 29131090 10.1159/000481177

[67] Ebert, D. D. et al. Internet- and mobile-based psychological interventions: Applications, efficacy, and potential for improving mental health. *Eur. Psychologist* **23**, 167–187 (2018).

[68] Weitzel, E. C. et al. E-mental-health und Digitale Gesundheitsanwendungen in Deutschland. *Der Nervenarzt* **92**, 1121–1129 (2021). 34608535 10.1007/s00115-021-01196-9

[69] Page, M. J. et al. The Prisma 2020 statement: An updated guideline for reporting systematic reviews. *BMJ.*10.1136/bmj.n71 (2021). 10.1136/bmj.n71 PMC8005924 33782057

[70] Harrer, M., Cuijpers, P., Furukawa, T. A. & Ebert, D. D. Doing meta-analysis with R 10.1201/9781003107347 (2021).

[71] Viechtbauer, W. Conducting meta-analyses inrwith themetaforpackage. *J. Stat. Softw.* **36**, (2010).

[72] Bischoff, C. et al. Wirksamkeit von Handheld-Gestütztem Selbstmanagement (e-coaching) in der rehabilitationsnachsorge. *Verhaltenstherapie* **23**, 243–251 (2013).

[73] Ebert, D. et al. Web-basierte rehabilitationsnachsorge nach stationärer Psychosomatischer Therapie (W-rena). *Die Rehab.* **52**, 164–172 (2013). 10.1055/s-0033-1345191 23761205

[74] Schmädeke, S. & Bischoff, C. Wirkungen smartphonegestützter Psychosomatischer Rehabilitationsnachsorge (eATROS) Bei depressiven Patienten. *Verhaltenstherapie* **25**, 277–286 (2015).

[75] Willems, R. A. et al. Short‐term effectiveness of a web‐based tailored intervention for cancer survivors on quality of life, anxiety, depression, and fatigue: Randomized controlled trial. *Psycho-Oncol.* **26**, 222–230 (2016). 10.1002/pon.4113 26988800

[76] Zwerenz, R. et al. Evaluation of a transdiagnostic psychodynamic online intervention to support return to work: A randomized controlled trial. *PLOS ONE* **12**, e0176513 (2017). 28481893 10.1371/journal.pone.0176513 PMC5421767

[77] Schlicker, S., Ebert, D. D., Middendorf, T., Titzler, I. & Berking, M. Evaluation of a text-message-based maintenance intervention for major depressive disorder after inpatient cognitive behavioral therapy. *J. Affect. Disord.* **227**, 305–312 (2018). 29132073 10.1016/j.jad.2017.10.047

[78] Zwerenz, R. et al. Improving the Course of Depressive Symptoms After Inpatient Psychotherapy Using Adjunct Web-Based Self-Help: Follow-Up Results of a Randomized Controlled Trial. *J. Med. Internet Res.* **21**, e13655 (2019). 31651403 10.2196/13655 PMC6838691

[79] Shaygan, M., Yazdani, Z. & Valibeygi, A. The effect of online multimedia psychoeducational interventions on the resilience and perceived stress of hospitalized patients with COVID-19: A pilot cluster randomized parallel-controlled trial. *BMC Psychiatry* **21**, 93 (2021). 33573631 10.1186/s12888-021-03085-6 PMC7877318

[80] Levis, M. et al. An implementation and effectiveness study evaluating Conflict Analysis in VA residential substance abuse services: Whole health informed self-guided online care. *EXPLORE* **18**, 688–697 (2022). 35219633 10.1016/j.explore.2022.02.005

